# Role of the Mitochondria in Immune-Mediated Apoptotic Death of the Human Pancreatic β Cell Line βLox5

**DOI:** 10.1371/journal.pone.0020617

**Published:** 2011-06-27

**Authors:** Yaíma L. Lightfoot, Jing Chen, Clayton E. Mathews

**Affiliations:** Department of Pathology, Immunology, and Laboratory Medicine, University of Florida College of Medicine, Gainesville, Florida, United States of America; University of Bremen, Germany

## Abstract

Mitochondria are indispensable in the life and death of many types of eukaryotic cells. In pancreatic beta cells, mitochondria play an essential role in the secretion of insulin, a hormone that regulates blood glucose levels. Unregulated blood glucose is a hallmark symptom of diabetes. The onset of Type 1 diabetes is preceded by autoimmune-mediated destruction of beta cells. However, the exact role of mitochondria has not been assessed in beta cell death. In this study, we examine the role of mitochondria in both Fas- and proinflammatory cytokine-mediated destruction of the human beta cell line, βLox5. IFNγ primed βLox5 cells for apoptosis by elevating cell surface Fas. Consequently, βLox5 cells were killed by caspase-dependent apoptosis by agonistic activation of Fas, but only after priming with IFNγ. This beta cell line undergoes both apoptotic and necrotic cell death after incubation with the combination of the proinflammatory cytokines IFNγ and TNFα. Additionally, both caspase-dependent and -independent mechanisms that require proper mitochondrial function are involved. Mitochondrial contributions to βLox5 cell death were analyzed using mitochondrial DNA (mtDNA) depleted βLox5 cells, or βLox5 ρ^0^ cells. βLox5 ρ^0^ cells are not sensitive to IFNγ and TNFα killing, indicating a direct role for the mitochondria in cytokine-induced cell death of the parental cell line. However, βLox5 ρ^0^ cells are susceptible to Fas killing, implicating caspase-dependent extrinsic apoptotic death is the mechanism by which these human beta cells die after Fas ligation. These data support the hypothesis that immune mediators kill βLox5 cells by both mitochondrial-dependent intrinsic and caspase-dependent extrinsic pathways.

## Introduction

Insulin-dependent, or Type 1 Diabetes Mellitus (T1D) results as a consequence of the specific autoimmune destruction of the pancreatic islet beta cells. While better understood in animal models, the exact progression to T1D in humans remains elusive, in part due to the limited human pancreatic samples available for research and the fact that the islets collected are obtained *postmortem* resulting in variable quality and functional capacity [1]. Consequently, animals that develop diabetes spontaneously and resemble the human form of the disease, like the NOD mouse and the BB-DP rat, as well as beta cell lines derived from murine sources, are heavily relied upon for a mechanistic understanding of the pathogenesis of the disorder [2].

Studies performed using animal models of T1D as well as primary human donor islets have proposed several direct and indirect mechanisms of beta cell destruction. For instance, in the NOD mouse, insulitis begins with the activation of macrophages and dendritic cells (DC) within the pancreatic islets. These resident specialized antigen-presenting cells locally produce chemokines and cytokines that recruit and activate autoreactive T and B lymphocytes [3]. Additionally, soluble mediators, such as cytokines and free radicals, both reactive nitrogen species (RNS) and reactive oxygen species (ROS) produced by the infiltrating immune cells and the beta cell themselves, can lead to beta cell death. In previous studies, IL-1β, IFNα, TNFα, and type 1 cytokines (IFNγ, TNFβ, IL-2, and IL-12) were found to correlate with destructive insulitis in the T1D prone NOD mouse and the BB rat [4]. Pancreatic samples from patients with T1D were also shown to contain the cytokines IFNα and IFNγ, TNFα-producing lymphocytes, as well as TNFα and IL-1β-expressing macrophages and DCs [5].

*In vitro* studies on the cytotoxicity of cytokines to beta cells suggest that individual proinflammatory cytokines can either enhance or inhibit beta cell insulin secretion depending on dose and length of exposure. However, when added in combinations, IL-1β, IFNγ, TNFα induce death and dysfunction of both human and rodent islets [6]. The impact of cytokines on mouse and rat islets is mainly through nitric oxide (NO)-mediated necrosis with minor contributions of apoptosis [6]–[16]. Studies reporting observations after exposing human islets to cytokines have been less clear, likely due to differences in experimental systems [17] as well as the health of the isolated human islets used [18], [19]. Taken together, it is rational to propose that when treated with cytokines, human islets die by both necrotic and apoptotic mechanisms.

Furthermore, these cytokines can either alone or in combination change the surface of islet cells, thereby enhancing the potential for immune surveillance by cytotoxic T cells. Predictably, molecules elevated by cytokines, such as MHC class I and Fas, have been correlated with destructive insulitis in both the mouse and human [20]. Beta cell surface remodeling by cytokines, combined with the fact that T1D is considered to be a T cell dependent disorder, imply that, *in vivo*, cytokines are responsible for providing an inflammatory environment conducive for T cell recognition and destruction of the insulin-producing cells. In this proinflammatory milieu, recognition of autoantigens by activated cytotoxic T cells (CTL) leads to direct beta cell lysis. CTL specific killing mechanisms that are thought to be involved in beta cell destruction include the Fas/FasL pathway and perforin/granzyme release. Also, analyses of pancreatic tissues of patients with T1D show Fas expression mainly in the remaining beta cells of the islets, and FasL expression in the infiltrating T lymphocytes [20], [21].

Most of these studies have been performed using both beta cells and CTLs derived from animal models. Yet, the appreciated genetic and immunopathologic differences between animal models of the disease and humans attest that diabetogenesis in humans could be distinctive and highlights the need for a human beta cell line that can be used for the study of death in the context of autoimmune-mediated destruction. In this report, we test the usefulness of a cell line derived from purified adult beta cells, βLox5 [22], in assays of beta cell death, as well as the mitochondrial contributions to human beta cell killing by immune effectors. We exposed βLox5 cells to direct killing by an activating human Fas antibody, CH11, in addition to indirect killing by the proinflammatory cytokines IFNγ and TNFα. The data presented show that similar to primary islets and beta cell lines derived from animals, βLox5 cells are killed after ligation of Fas by caspase-dependent apoptosis, whereas these cells die by caspase-dependent and –independent apoptosis together with necrosis after incubation with TNFα and IFNγ. Importantly, βLox5 cells depleted of their mitochondrial DNA were resistant to proinflammatory cytokine-induced killing, implicating a role for mitochondria associated cell death mechanisms in the progression to T1D in humans.

## Results

### Agonistic activation of Fas kills βLox5 cells by caspase-dependent apoptosis

**Figure 1:**
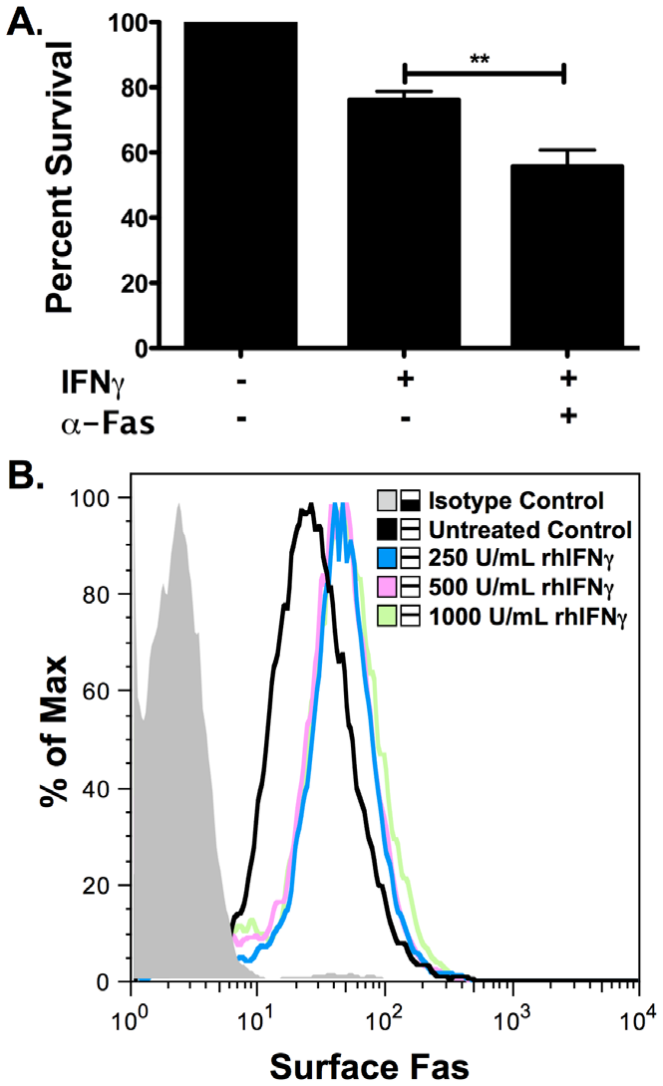
βLox5 cells are susceptible to α-Fas monoclonal antibody after rhIFNγ priming. **A**. βLox5 cells were treated with rhIFNγ alone or the combination of α-Fas antibody clone CH-11 (0.5 mg/mL) and rhIFNγ (1000 U/mL) for 48 h. Viability was measured by the MTT assay. ** denotes statistical significance, p<0.005. **B**. Overnight priming of βLox5 cells with rhIFNγ increases the expression of surface Fas similarly with 250 U/mL (light gray line), 500 U/mL (gray line), or 1000 U/mL (dark gray line) of rhIFNγ when compared to untreated control cells (black line).

**Figure 2:**
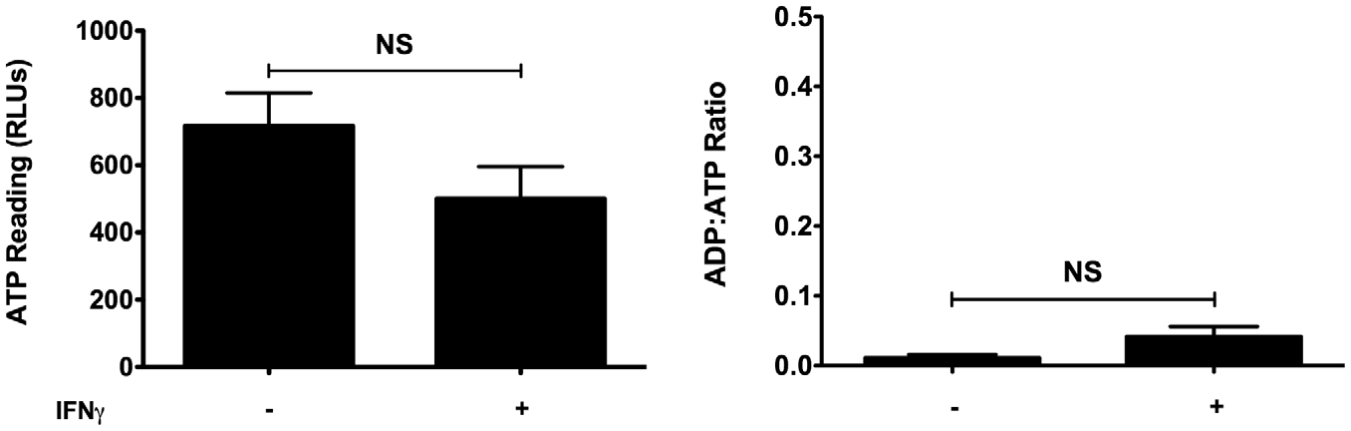
rhIFNγ alone causes arrested proliferation. βLox5 cells were treated with rhIFNγ (1000 U/mL) for 48 h. Cell death profile was analyzed by the ApoGlow assay. NS denotes no statistical difference.

βLox5 cells were incubated for 48 hours with α-Fas monoclonal antibody (CH11) alone or in combination with rhIFNγ. The combination of CH11 and rhIFNγ induced death of the βLox5 cells (**Fig. 1A**), while neither CH11 nor rhIFNγ were alone effective. rhIFNγ was required for Fas induced cell death as it increased Fas expression on the cell surface, even at the lowest level of rhIFNγ tested (**Fig. 1B**). Because βLox5 cells treated with rhIFNγ had a reduction in absorbance during the MTT assay, we performed the ApoGlow assay to distinguish between inhibition of proliferation and cell death. rhIFNγ treatment of βLox5 cells lowered ATP levels with little or no change in the ADP to ATP ratios corresponding with arrested proliferation (**Fig. 2**).

**Figure 3:**
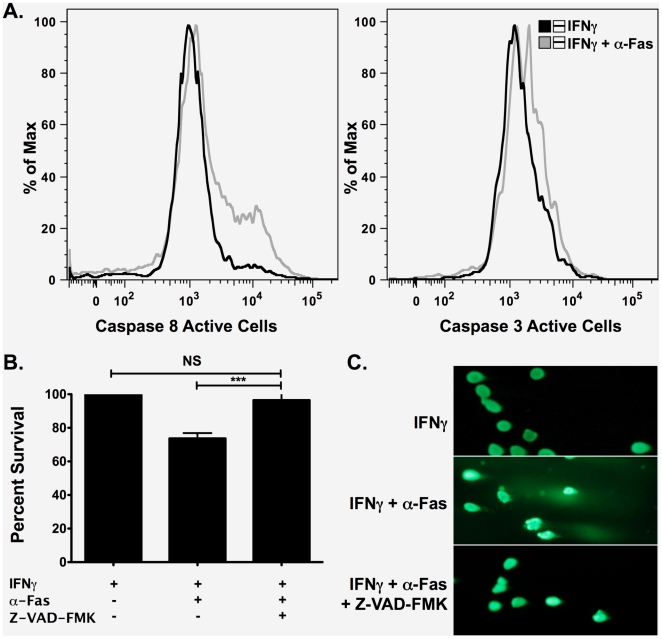
Fas-induced killing is caspase-dependent. **A**. βLox5 cells were primed overnight with rhIFNγ (1000 U/mL) then left untreated or treated with α-Fas antibody clone CH-11 (0.5 mg/mL) for an additional 24 h before the activities of Caspases 8 and 3 were measured by FACS analysis. Increased Caspase 8 and 3 activities were noted after only 24 h of Fas stimulation. A representative plot is shown. **B & C**. βLox5 cells were treated with rhIFNγ alone or the combination of α-Fas antibody clone CH-11 (0.5 mg/mL) and rhIFNγ (1000 U/mL) for 48 h with and without pan-caspase inhibition with Z-VAD-FMK (50 µM x 2). Viability was measured by the MTT assay (**B**). *** denotes statistical significance with a P value <0.0001. NS denotes no statistical difference. DNA damage after treatment with and without caspase inhibition was assessed by the Comet Assay (**C**).

To determine the mechanism of α-Fas-induced killing of βLox5 cells, caspase activity was assayed. Caspases 8 and 3 were shown to be active after only 24 hours of treatment (**Fig. 3A**). Pan-caspase inhibitor Z-VAD-FMK (50 µM) was added to cells 1 hour prior to and again 24 hours after initiation of rhIFNγ and CH11 treatment. When compared to rhIFNγ control samples, caspase inhibition increased cell survival to control levels (**Fig. 3B**) and eliminated DNA damage (**Fig. 3C**). These data clearly implicate caspase-induced apoptosis as the necessary pathway in Fas-mediated killing of βLox5 cells.

### Proinflammatory cytokine-induced killing of βLox5 cells occurs through caspase-dependent and –independent apoptosis and necrosis

**Figure 4:**
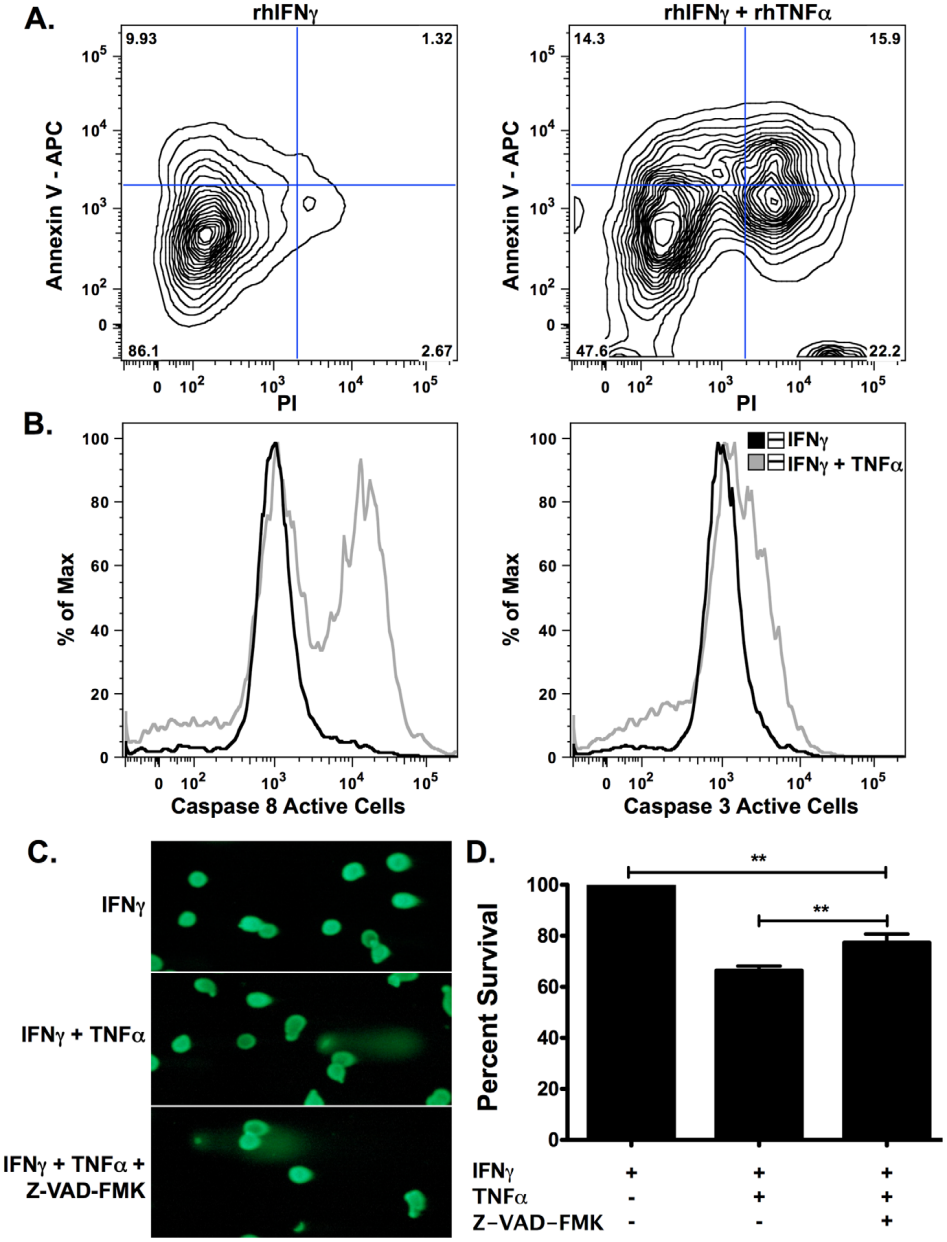
Cytokine-induced cell death is partially caspase-dependent. **A, B, C & D.** βLox5 cells were treated with the combination of rhTNFα (2000 U/mL) and rhIFNγ (1000 U/mL) for 48 h with and without pan-caspase inhibition with Z-VAD-FMK (50 µM x 2). The cell death profile (**A**) as well the activities of Caspases 8 and 3 (**B**) were measured by FACS analysis. A representative plot is shown. DNA damage and death were assessed via the Comet Assay (**C**) and the MTT Assay (**D**), respectively. ** denotes statistical significance with a P value <0.005.

βLox5 cells were also susceptible to proinflammatory cytokine-mediated cell death. Treatment of these cells with the combination of rhTNFα and rhIFNγ for 48 hours caused significant killing (**Fig. 4**); however, neither of these cytokines alone was sufficient to kill βLox5 cells. Similar to NOD derived NIT-1 beta cells, the addition of IL-1β to the combination is dispensable [23], [24]. Consequently, NO was not detected in the supernatant when measured indirectly via the Greiss Reaction. In addition, Heat Shock Proteins (HSPs), specifically HSP70 and HSP27 (**Fig. S1**), which have been shown to protect beta cells against proinflammatory cytokine induction of Nitric Oxide Synthase (iNOS) and subsequent production of NO [8], [9], [15], [25], [26], were expressed in untreated βLox5 cells and both HSP27 and HSP70 were significantly upregulated by rhIFNγ and the combination of rhIFNγ and rhTNFα. Because rhIFNγ was shown to inhibit proliferation of βLox5 cells, we tested the effects of rhTNFα on the proliferation of these cells using a tritiated thymidine (^3^H-TdR) incorporation assay. In contrast to rhIFNγ, rhTNFα-treated cells (1000 U/mL or 5000 U/mL) incorporated the same amount of ^3^H-TdR after 24 and 48 hours as untreated cells (Data Not Shown).

**Figure 5:**
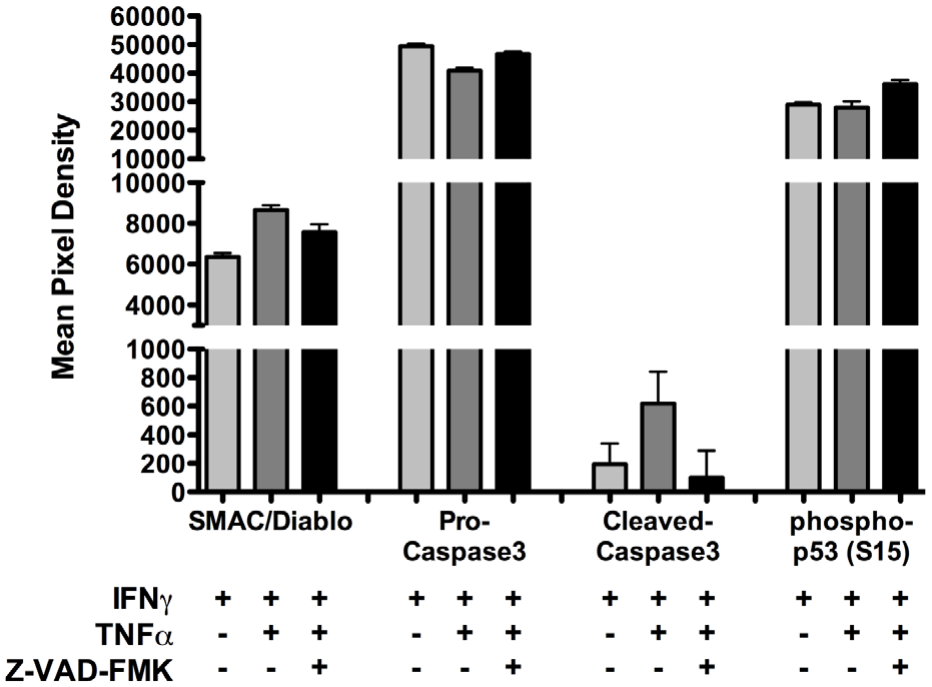
Apoptosis and DNA repair protein expression of cytokine-treated βLox5 cells. βLox5 cells were treated with the combination of rhTNFα (2000 U/mL) and rhIFNγ (1000 U/mL) for 24 h with and without pan-caspase inhibition with Z-VAD-FMK (50 µM). The Proteome Profiler Human Apoptosis Array Kit was used for protein detection. Proteins of interest are shown.

Treatment of βLox5 cells with rhIFNγ and rhTNFα resulted in activation of Caspases 8 and 3 (**Fig. 4B**). Confirmed pan-caspase inhibition failed to completely prevent death (**Fig. 4C, D**), suggesting that cytokines kill these cells by multiple pathways. Accordingly, flow cytometric and Comet assay analyses of cytokine-induced βLox5 killing indicated that these cells die by apoptosis and necrosis (**Fig. 4A, C**). Although caspase inhibition significantly improved βLox5 viability when measured by the MTT assay, levels did not reach rhIFNγ control (**Fig. 4D**) and DNA damage was still observed (**Fig. 4C**). Protein levels of phosphorylated p53 (S15) increased with pan-caspase inhibition (**Fig. 5**), indicating an enhanced effort to repair DNA damage. Cell death in βLox5 cells was preceded by an increase in the pro-apoptotic protein SMAC/Diablo (**Fig. 5**). SMAC/Diablo contributes to the caspase cascade by binding to inhibitors of apoptosis (IAPs), such as XIAP [27]. As a result, pro-caspase 3 levels decreased, while cleaved caspase 3 increased (**Fig. 5**).

**Figure 6:**
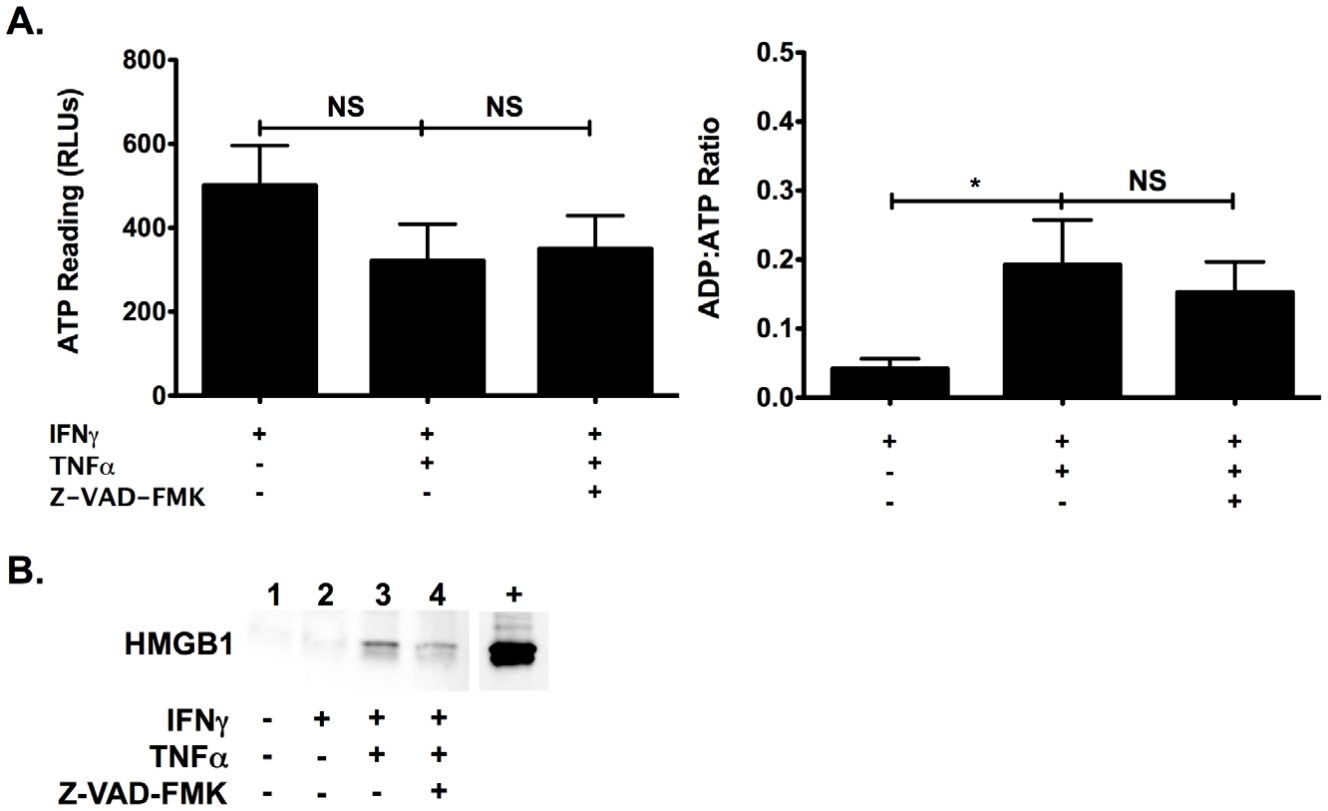
βLox5 cells die by apoptosis and necrosis after cytokine treatment with and without pan-caspase inhibition. **A & B.** βLox5 cells were treated with the combination of rhTNFα (2000 U/mL) and rhIFNγ (1000 U/mL) for 48 h with and without pan-caspase inhibition with Z-VAD-FMK (50 µM x 2). Viability was analyzed by the ApoGlow Assay (**A**). * denotes statistical significance with a P value <0.05. NS denotes no statistical difference. The supernatant was analyzed for passive HMGB1 release (**B**). **+**: βLox5 cell lysate/positive control, **1**: Untreated Control, **2**: rhIFNγ, **3**: rhIFNγ + rhTNFα, **4**: rhIFNγ + rhTNFα + Z-VAD-FMK.

Apoptotic and necrotic βLox5 cell death after cytokine treatment and caspase inhibition were further analyzed by the ApoGlow assay and detection of passively released High Mobility Group 1 (HMGB1), which has been demonstrated to only be released during primary necrosis [28]–[30]. The ApoGlow assay showed signatures of apoptosis, including reductions in ATP levels of cytokine treated groups with and without pan-caspase inhibition compared to rhIFNγ controls, and significant increases in the ADP to ATP ratios that did not differ between the cells treated with rhTNFα and rhIFNγ (**Fig. 6A**). These results indicate that the improved viability after pan-caspase inhibition measured by the MTT assay was not biologically significant and apoptosis was still taking place. Necrosis was confirmed by the presence of HMGB1 only in the supernatant of cytokine-treated βLox5 cells, compared to untreated and rhIFNγ control cells (**Fig. 6B**).

**Figure 7:**
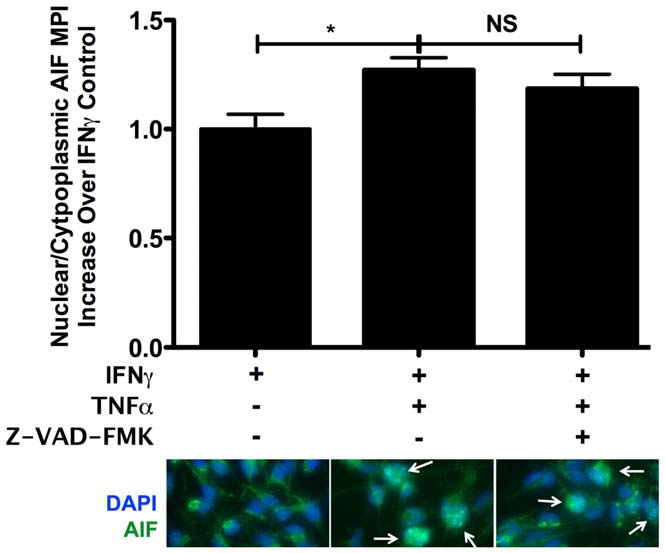
Cytokine treatment of βLox5 promotes nuclear translocation of Apoptosis Inducing Factor. βLox5 cells were treated with the combination of rhTNFα (2000 U/mL) and rhIFNγ (1000 U/mL) for 48 h with and without pan-caspase inhibition with Z-VAD-FMK (50 µM x 2). Immunofluorescence analysis shows increased AIF translocation to the nucleus in cytokine treated cells. Representative images are shown. White arrows indicate cells with high nuclear AIF staining. *denotes statistical significance with a P value <0.05. NS denotes no statistical difference.

To examine the contribution of caspase-independent, proapoptotic molecules, Apoptosis Inducing Factor (AIF) localization was visualized by immunofluorescence. Compared to rhIFNγ controls, more AIF was present in the nucleus of rhIFNγ and rhTNFα-treated cells (**Fig. 7**). This suggests that AIF is involved in the demise of βLox5 cells that occurs in the presence of proinflammatory cytokines. Inhibition studies to further understand the mechanisms of βLox5 cell death showed that these do not depend on Bax translocation, or Cathepsin B activity (Data Not Shown).

### Mitochondrial-DNA deficient βLox5 cells are resistant to cytokine killing but sensitive to Fas

**Figure 8:**
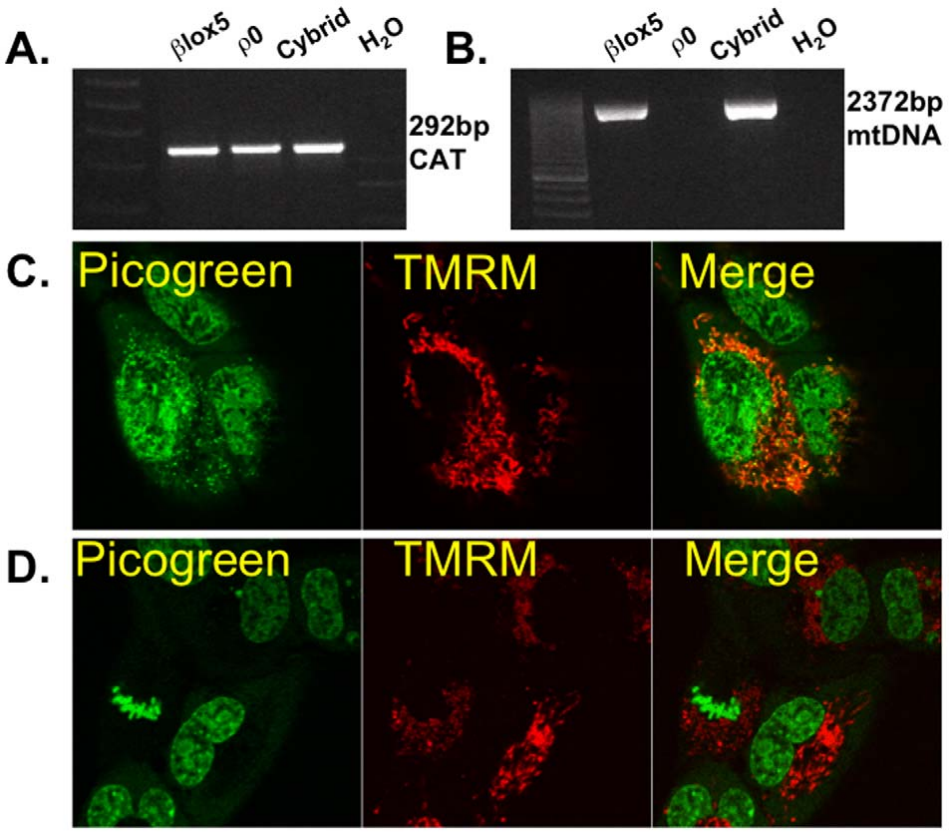
Confirmation of mtDNA depletion in βLox5 ρ^0^ cells. **A**. PCR primers specific for a segment of the Catalase gene (CAT) were used as a positive control. Genomic DNA from both EtBr (100 ng/mL) treated and untreated cell cultures exhibited robust amplification with the CAT primer pair (product length: 292 bp). **B**. Using primers that are specific for the human mtDNA we were unable to amplify mtDNA from the EtBr treated cells, while the untreated cells produced a band of appropriate size (product length: 2372 bp). **C**. Confocal images of untreated βLox5 cells using the fluorescent probes picogreen (Green-DNA) and TMRM (Red-mitochondrial membrane potential). Untreated cells exhibit a co-localization (Orange) of these dyes in the cytoplasm. **D**. Confocal images of EtBr treated (100 ng/mL) βLox5 cells (βLox5 ρ^0^) using the fluorescent probes picogreen (Green-DNA) and TMRM (Red-mitochondrial membrane potential). βLox5 ρ^0^ cells exhibit mitochondrial membrane potential but no cytoplasmic positivity for picogreen.

To examine the role of the mitochondria in cytokine-induced killing of βLox5 cells, the cell line was depleted of mitochondrial-DNA (βLox5 ρ^0^) using low levels of EtBr. PCR and confocal imaging of the cells confirmed successful depletion of the mtDNA (**Fig. 8**). Treated and untreated βLox5 cells amplified primers specific for the nuclear-encoded human Catalase gene (**Fig. 8A**), but only untreated cells amplified mtDNA-specific primers (**Fig. 8B**). A mitochondrial marker, TMRM, was used to identify the mitochondria, and picogreen was used as a DNA dye to identify cytoplasmic DNA. Co-localization of the red TMRM and green picogreen in the untreated cells was indicative of the presence of DNA in the mitochondria (**Fig. 8C**). However, EtBr treated cells did not co-localize the fluorescent dyes (**Fig. 8D**).

**Figure 9:**
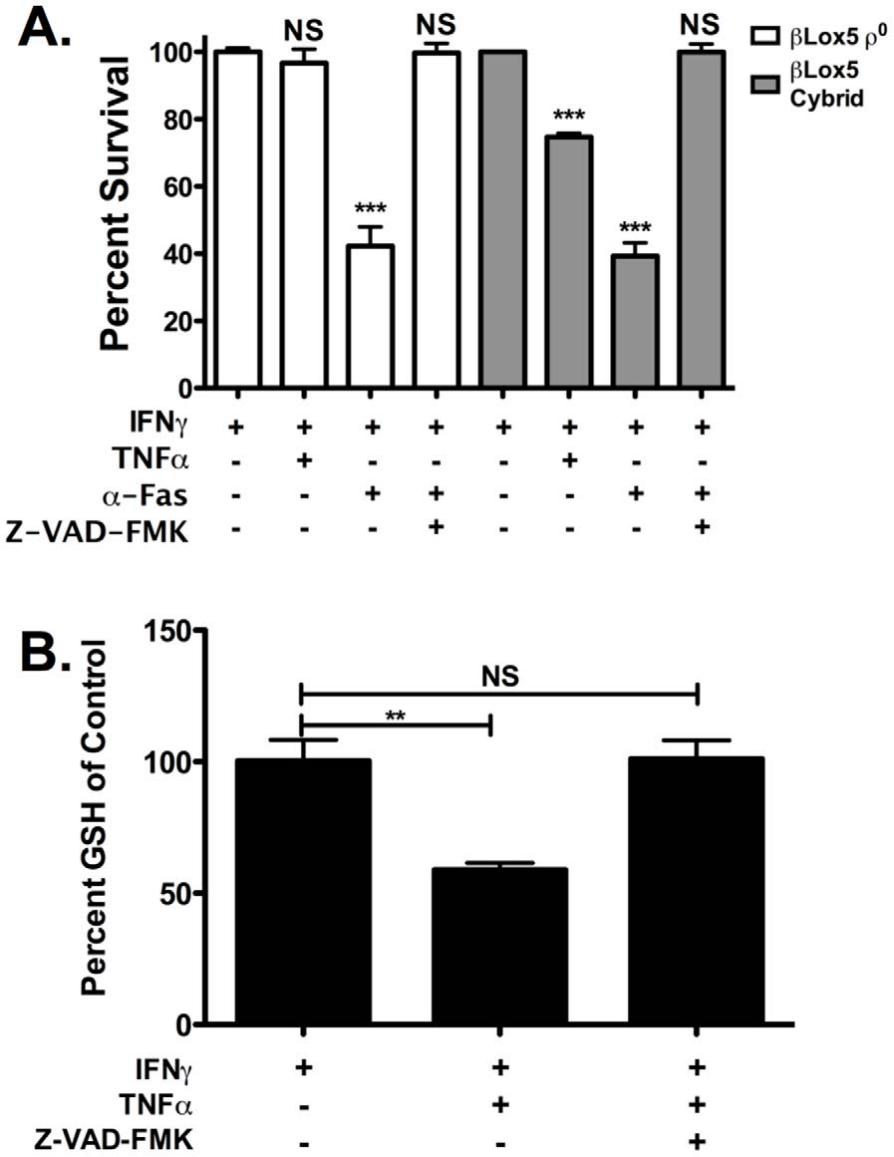
Functional mitochondria are required for cytokine killing of βLox5. **A**. βLox5 ρ^0^ cells (empty bars) and βLox5 Cybrid cells (gray bars) were treated with rhTNFα (2000 U/mL) and rhIFNγ (1000 U/mL) or with CH-11 (0.5 mg/mL) and rhIFNγ (1000 U/mL) for 48 h. Z-VAD-FMK (50 µM x 2) was used to inhibit caspase activity. Viability was measured by the MTT assay. Only mtDNA sufficient cells were killed by cytokines. *** denotes statistical significance with a P value <0.0001 compared to rhIFNγ control. NS denotes no statistical difference when compared to rhIFNγ control. **B**. Changes in GSH levels were measured after a 24 h incubation period with rhTNFα (2000 U/mL) and rhIFNγ (1000 U/mL), with and without pan-caspase inhibition. ** denotes statistical significance with a P value <0.01 compared to rhIFNγ control.

Viability of βLox5 ρ^0^ cells after cytokine treatment was measured by the MTT assay and confirmed by FACS analysis of PI exclusion. βLox5 ρ^0^ cells were found to be resistant to cytokine-mediated cell death but sensitive to Fas-induced killing (**Fig. 9A**), supporting activation of the extrinsic pathway by Fas versus the intrinsic pathway by proinflammatory cytokines. mtDNA sufficient βLox5 Cybrid cells were sensitive to Fas ligation and add-back of mtDNA resulted in regained susceptibility to proinflammatory cytokines (**Fig. 9A**). Pan-caspase inhibition was able to prevent Fas-induced cell death in ρ^0^ and Cybrid cells (**Fig. 9A**). Because the mitochondrial electron transport chain is the main source of ROS in cells, we analyzed the redox state of the parental cell line, βLox5, after cytokine treatment. Reductions in available GSH were observed after 24 h of cytokine treatment (**Fig. 9B**) when no changes in cell survival have been noted.

## Discussion

A human beta cell line that can be expanded and maintained indefinitely would be a useful tool for advancing our understanding of the autoimmune pancreatic beta cell destruction that precedes Type 1 diabetes development in man. Such a model cell would also provide an *in vitro* system to test pharmacological inhibitors or genetic manipulations intended to block killing by autoimmune effectors, or invasively study the impact of immunosuppressive agents, hyperglycemia, or hyperlipedemia. To date, there have been publications detailing the production of six human derived beta cell lines. These lines are NAKT-15 [31], CM and HP62 [32]–[40], NES Y2 [41], TRM-6 [42] and βLox5 [22], [42]–[46]; however, only βLox5 is readily available to the scientific community. We sought to determine the value of this already established, human pancreatic beta cell-derived line, βLox5, in studies aimed at elucidating the role of mitochondria in human beta cell death induced by immune insults.

The Fas/FasL pathway has been associated with the development of T1D in animal models and in humans [20], [21], [47]–[49]. Although autoreactive T cell clones from transgenic mice lysed Fas-deficient islets, presumably due to perforin release, perforin-deficient T cells had similar diabetogenic potential as the wild type clones when transferring disease to immunodeficient NOD mice [20], [50]. These findings suggest that redundant mechanisms eliminate beta cells during the autoimmune attack. To mimic direct killing by diabetogenic effectors, we incubated the cell line with an activating α-Fas monoclonal antibody for 48 h. The 48 h treatment period was chosen to obtain a significant amount of cell death while still having the required cell numbers to perform functional assays, such as measuring caspase activity in apoptotic and live cells. In addition, because βLox5 cells proliferate well, longer incubation times in 12-well plates leads to death even in untreated cells.

Similar to primary islets from human and mouse as well as rodent derived beta cell lines, βLox5 cells required rhIFNγ priming for sufficient surface Fas expression and subsequent ligation by the antibody (**Fig. 1**), supporting the role of proinflammatory cytokines in providing an environment favorable for cell killing [51]. Moreover, Fas-dependent apoptosis in βLox5 cells was found to be caspase mediated (**Fig. 3**). mtDNA deficient βLox5 ρ^0^ cells, which are deficient in the electron transport chain subunits of Complexes, I, III, and IV that are encoded by the mtDNA, were sensitive to Fas ligation due to the activation of caspases (**Fig. 9A**). This is in accordance with the extrinsic type I model of Fas-mediated apoptosis that proceeds independent of the mitochondria [52], and with Fas-mediated killing mechanisms previously identified in the NIT-1, NOD insulinoma cells, and primary NOD islets [53], [54]. These results indicate that βLox5 cells are susceptible to direct killing by immune effectors and die by a relevant pathway in T1D.

Proinflammatory cytokine exposure of primary rat or mouse pancreatic islets as well as the RIN and INS1 beta cell lines, established from rat, results in functional inhibition and death that is highly dependent upon the production of NO [12] with small contributions of apoptosis only after long-term culture with cytokines [13]. In mouse beta cell lines, killing due to IFNγ, TNFα and IL-1β treatment is less dependent on NO production and in some cell lines death is NO independent [55], [56]. In human islets, timing of treatment as well as cytokine combination and dose are critical [11], [17]. To test if βLox5 cells are also vulnerable to proinflammatory cytokines, we cultured the cells with rhIFNγ, rhTNFα and rhIL-1β individually or in combination. We found that the combination of rhIFNγ and rhTNFα led to the most significant level of βLox5 cell death by both apoptosis and necrosis (**Fig. 4**). Cytokines promoted both necrosis and caspase-dependent apoptosis of βLox5 cells that was independent of NO, potentially due to the presence of HSP27 and HSP70 (**Fig. S1**), confirming that proinflammatory cytokines can activate a range of pro-death mechanisms in beta cells [5], [10], [13], [17], [53]–[55], [57]–[63].

Beta cell mitochondria play a key role in insulin secretion [64] and may be important in beta cell death. Apoptosis-inducing stimuli result in the mitochondrial membrane permeability transition (PT) that leads to the release of Cytochrome c (Cyt c) and other proapoptotic molecules. PT and Cyt c release generally precede the disruption of mitochondrial inner membrane potential (ΔΨm) and mitochondrial function [65]. In addition, mitochondrial release of proapoptotic molecules potentiates the activation cascade of caspases [66]. Although caspases are involved in the killing of βLox5 cells by proinflammatory cytokines (**Figs. 4** **&** **5**), other pathways are also implicated, as the pan-caspase inhibitor, Z-VAD-FMK, failed to completely prevent death in these cells (**Figs. 4** **&** **6**). Necrosis was shown to contribute to βLox5 cytokine-induced death (**Figs. 4** **&** **6**) and could have accounted for the above results; however, DNA damage, which is indicative of apoptosis, was still observed with pan-caspase inhibited cells (**Figs. 4** **&** **5**). Therefore, caspase-independent mechanisms of apoptosis were investigated. Apoptosis Inducing Factor (AIF) is a caspase-independent, proapoptotic molecule that acts through its release from the mitochondria and subsequent translocation to the nucleus, where it binds DNA and causes chromatin condensation [67], [68]. In order to determine if AIF is involved in cytokine killing of βLox5 cells, treated cells were visualized for AIF localization. The ratio of nuclear localized AIF to cytoplasmic AIF was significantly increased in cytokine-treated cells (**Fig. 7**). This difference persisted in Z-VAD-FMK pre-treated βLox5 cells, supporting a role for AIF in rhIFNγ and rhTNFα cytotoxicity.

Other caspase-independent pro-apoptotic pathways were studied for their role in cytokine-mediated βLox5-cell death. Cathepsin B has been shown to contribute to TNFα-induced apoptosis in other cell types [69]; however, Cathepsin B inhibition with CA 074 failed to rescue βLox5 survival after cytokine treatment. In fact, at the published concentration of 20 µM [69], CA 074 exacerbated cytokine killing and caspase activation (Data Not Shown). At the highest non-toxic concentration of 5 µM, CA 074 did not increase survival. Recently, Bax-dependent mitochondrial permeabilization was identified as a pro-apoptotic signal in human islets after cytokine treatment [17]; nonetheless, preventing Bax translocation into the mitochondria was insufficient to prevent death in βLox5 cells (Data Not Shown).

mtDNA deficient βLox5 ρ^0^ cells were not killed by rhIFNγ and rhTNFα treatment but mtDNA sufficient βLox5 Cybrid cells were sensitive to cytokine-induced cell death (**Fig. 9A**). This is consistent with a recent report that demonstrated the intrinsic apoptosis was activated during cytokine treatment of human islets [17]. Therefore, because we found that functional mitochondria are required for IFNγ and TNFα killing and mitochondria are a major source of ROS, we tested the cells for signs of oxidative stress. GSH levels after cytokine treatment indicated oxidative stress in these cells (**Fig. 9B**), demonstrating that cytokine treatment tilts the redox balance towards oxidation, likely due to increased ROS production.

The susceptibility of isolated human islets to killing *in vitro* by proinflammatory cytokines has been the focus of significant hypothesis testing, while mechanisms of Fas killing of human islets has been less intense [70]. Post-mortem histological analysis of pancreas from patients with T1D have demonstrated that within the insulitis, CD8^+^ T cells express cell surface FasL suggesting a role for Fas in beta cell destruction during disease development [71]. Information on how Fas kills islets has been derived from studies using mouse islets with no clear published mechanism using human islets. Mouse studies indicated Fas activated caspases. The enclosed studies are the first demonstration of a mechanism for Fas mediated apoptosis of human beta cells, and clearly indicate that Fas activates the extrinsic pathway for apoptosis in βLox5 cells (**Figs. 3** **&** **9**). It remains to be investigated whether this is a shared mechanism with primary human islets.

The ultimate effector molecule resulting from a cytokine attack on primary islets is NO [11]. In contrast to primary human islets, βLox5 cells do not produce NO when exposed to the combination of IFNγ, TNFα and IL-1β. In the absence of NO production, IFNγ, TNFα and IL-1β can activate a range of pro-death pathways, including caspase-dependent apoptosis and necrosis, in isolated primary human islets [17], [70]. βLox5 cells undergo both necrosis and caspase-dependent and –independent apoptosis after treatment with rhIFNγ and rhTNFα. However, IL-1β is required to kill primary human islets yet, its addition is superfluous for killing of βLox5 cells. Therefore, this cell line is likely unsuitable as a model to use in studies of cytokine-induced iNOS-mediated beta cell killing.

In summary, βLox5 cells are a partially dedifferentiated beta cell line that produces a lower level of insulin than primary islets and has blunted glucose stimulated insulin secretion. This cell line and its derivative line, βLox5 ρ^0^, have been established as unlimited sources of human beta cells, that are inappropriate to study mechanisms of beta cell function; however, they can be used for the study of autoimmune beta cell apoptosis as well as mitochondrial contributions to cell death. These cells are primed by proinflammatory cytokines for Fas-induced caspase-dependent apoptosis via the extrinsic pathway and are susceptible to cytokine-mediated apoptosis and necrosis through mitochondrial mechanisms that are both caspase-dependent and –independent. We conclude that these cells will likely be beneficial for analyzing direct methods of killing employed by autoimmune effector cells.

## Materials and Methods

### Cell Line and Reagents

The βLox5 cell line was kindly provided by Dr. Fred Levine (Sanford Children's Health Research Center, Sanford-Burnham Medical Research Institute, La Jolla, CA). βLox5 cells were maintained in low glucose (1 mg/mL) DMEM (Cellgro, Manassas, VA), supplemented with 10% FBS (HyClone, Fisher Scientific, Pittsburgh, PA), 1% MEM non-essential amino acids (Cellgro), 1% penicillin-streptomycin (Gemini Bio-Products, West Sacramento, CA) solution, 0.02% BSA (Sigma, St. Louis, MO) and 15 mM HEPES (Cellgro) (VC-DMEM). Recombinant human IFNγ was obtained from BD Biosciences (San Jose, CA). Recombinant human TNFα and recombinant IL-1β were purchased from R&D Systems (Minneapolis, MN). Fas agonistic antibody (Clone CH11) was purchased from Millipore (Temecula, CA). A monoclonal antibody to HMGB1 was acquired from Abcam Inc. (Cambridge, MA). An antibody to hCD95 (Fas)-PE-Cy5 and the isotype control were purchased from BD Biosciences. Annexin V-APC was purchased from Invitrogen (Carlsbad, CA).

### Generation of βLox5 ρ^0^ Cells and βLox5 Cybrid Cells

βLox5 ρ^0^ cells were cultured in high glucose (4.5 mg/mL) DMEM (Cellgro) supplemented with 10% FBS (HyClone, Fisher Scientific), 1 mM sodium pyruvate (Sigma), 50 mg/L Uridine (Sigma), and 1% penicillin-streptomycin (Gemini Bio-Products). mtDNA was depleted by culturing cells in the above medium supplemented with 100 ng/ml Ethidium Bromide (EtBr) for 6 months. Depletion of mtDNA was confirmed by: 1) PCR; 2) confocal microscopy imaging; 3) failure of βLox5 ρ^0^ cells to survive in pyruvate- and uridine-free medium.

βLox5 Cybrid cells were generated as described before [72]. Briefly, cybrid cells were made by fusion of βLox5 ρ^0^ cells with mtDNA donor platelets from a healthy individual under the presence of 50% (W/V) polyethylene glycol 1500 (Roche). Cells were cultured in the medium for βLox5 ρ^0^ cells during the first 3 days after the fusion and then in selective medium (uridine and pyruvate-free DMEM supplemented with 10% dialyzed FBS, Penicillin and Streptomycin). After selection for 3 weeks, surviving cybrid cells were cultured in DMEM for βLox5, as described above, without pyruvate and uridine. Cybrid cells were cloned using cloning cylinders (Corning, Corning, NY) when visible colonies appeared in the culture.

#### Cell Death Assays

βLox5, ρ^0^ and Cybrid cells were seeded in twelve-well Corning Costar culture plates (Fisher Scientific) at a density of 5×10^4^ cells per well in a total volume of 500 µL and allowed to adhere for 48 hours. The cells were then incubated with rhTNFα (2000 U/mL) and rhIFNγ (1000 U/mL) for 48 hours. βLox5, ρ^0^ and Cybrid cells were also cultured in the presence of α-Fas activating antibody CH11 (0.5 mg/mL) with and without rhIFNγ (1000 U/mL). Cell viability was examined using the MTT assay [73], [74], propidium iodide (PI) uptake, and externalization of phosphatidylserine (PS) by Annexin V-APC staining.

Percent cell survival after cytokine or α-Fas antibody treatment was measured by determining the ability of the live cells to reduce yellow MTT, 3-(4,5-Dimethylthiazol-2-yl)-2,5-diphenyltetrazolium bromide (Sigma), to insoluble purple formazan crystals. The cells were treated with MTT solution [5 mg/mL in phosphate buffered saline (PBS)] for 2 hours at 37°C, 5% CO_2_. Excess solution was removed and the formazan crystals were then resuspended in acid isopropanol (0.04 N HCl in isopropanol). The optical density of the product was measured at a wavelength of 560 nm and background subtracted at 670 nm.

βLox5 cells were analyzed on a BD LSR-Fortessa flow cytometer using the BD FACSDiva software (BD Biosciences) and FlowJo flow cytometry analysis software (Tree Star, Inc., Ashland, OR). Cellular apoptosis was determined by double staining with PI and Annexin V-APC, while necrotic cells were identified as PI single positive cells. In addition, Trevigen's CometAssay kit (Trevigen Inc, Gaithersburg, MD) was used to evaluate DNA damage in treated and untreated βLox5 cells based on DNA tail shape and migration pattern. The Proteome Profiler Human Apoptosis Array Kit (R&D Systems) was utilized to measure the expression of proteins involved in apoptosis and DNA repair.

Passive release of high-mobility group box 1 (HMGB1) protein by necrotic cells was determined Western Blot. Briefly, βLox5 cells were treated as described and the supernatant of each well was removed without disturbing the attached cells. The supernatant was concentrated using an ultracentrifuge (100,000 x g for 25 minutes), separated by SDS-PAGE, and then transferred to a nitrocellulose membrane (Bio-Rad Laboratories, Hercules, CA). HMGB1 was detected by chemiluminescence (SuperSignal West Pico Chemiluminescent Substrate Kit, Thermo Scientific, Waltham, MA) according to the manufacturer's instructions.

### Nitric Oxide Detection

The amount of nitric oxide (NO) released by βLox5 cells after cytokine treatment was indirectly measured using the Griess Reaction as previously described [14]. The optical density was read using a SpectraMax M5 plate reader (Molecular Devices, Sunnyvale, CA).

### Oxidative Stress Analysis

Glutahione (GSH) levels were detected and quantified with the GSH-Glo Glutathione Assay (Promega, Madison, WI) after a 24 hour-incubation with rhTNFα (2000 U/mL) and rhIFNγ (1000 U/mL).

### Caspase Activity and Inhibition Assays

Caspase-8 and caspase-3 activities were measured using a commercially available caspase detection kit (Cell Technology, Inc, Palo Alto, CA) according to the manufacturer's instructions. Briefly, caspase-8 (FAM-LETD-FMK) or caspase-3 (FAM-DEVD-FMK)-specific carboxyfluorescein (FAM) labeled peptide fluoromethyl ketone (FMK) caspase inhibitors were incubated with 48 hours cytokine-treated, 24 hours and 48 hours α-Fas-treated, or untreated control βLox5 cells for 1 hour at 37°C. Cells containing bound inhibitor were analyzed by flow cytometry on the FL1 channel. In some cases, the cells were treated with 50 µM of the pan-caspase inhibitor (Z-VAD-FMK), purchased from Calbiochem (San Diego, CA), for 1 hour prior to treatment. Z-VAD-FMK was also added after 24 hours of incubation to maintain caspases inactive. Pretreatment for 1 hour with the specific inhibitor CA 074 (20 µM) (Sigma) was used to inhibit the lysosomal protease Cathepsin B. Bax translocation into the mitochondria was inhibited with 100 µM of the peptide V5 (Calbiochem).

### Flow Cytometry

Cytokine-treated and untreated βLox5 cells were analyzed for Fas surface expression by standard flow cytometry techniques. In brief, βLox5 cells were treated with increasing concentrations of rhIFNγ (250, 500, and 1000 U/mL) for 48 hours, stained for 1 hour at 4°C and washed to remove excess unbound antibody before analysis.

### Immunofluorescence

βLox5 cells were incubated with cytokines as described. Cells were fixed with 2% paraformaldehyde (PFA) for 10 minutes at room temperature (RT), permeabilized with 100% ice-cold methanol for 10 minutes, then blocked with 10% Normal Goat Serum (NGS) for 40 minutes at RT with single PBS washes between each step and two washes before adding the antibody. βLox5 cells were conjugated with AIF antibody (R&D Systems) for 1 hour at 37°C, washed 3 times, then stained with FITC conjugated anti-rabbit IgG (1∶200) for 60 minutes in the dark. Before visualization, cells were washed and slides were covered using 4′,6-diamidino-2-phenylindole (DAPI)-containing mounting medium. A Zeiss Axioskop Microscope was used to visualize and image the cells. Images were analyzed using ImageJ/Fiji (National Institute of Health).

### Statistical analysis

Unless stated otherwise, data are shown as mean ± SEM. Significance was determined by a T-test for two group comparisons (GraphPad Prism 5.02 San Diego, CA).

## Supporting Information

Figure S1 **Cytokine treatment of βLox5 cells induces the expression of Heat Shock Proteins.** βLox5 cells were treated with the combination of rhTNFα (2000 U/mL) and rhIFNγ (1000 U/mL) for 24 h with and without pan-caspase inhibition with Z-VAD-FMK (50 µM). The Proteome Profiler Human Apoptosis Array Kit was used for protein detection.
(TIF)
