# Supplementary figures and images for: Role of the Mitochondria in Immune-Mediated Apoptotic Death of the Human Pancreatic β Cell Line βLox5
Source: PLoS One. 2011 Jun 27;6(6):e20617. doi: 10.1371/journal.pone.0020617 (PMC3124469; doi:10.1371/journal.pone.0020617)

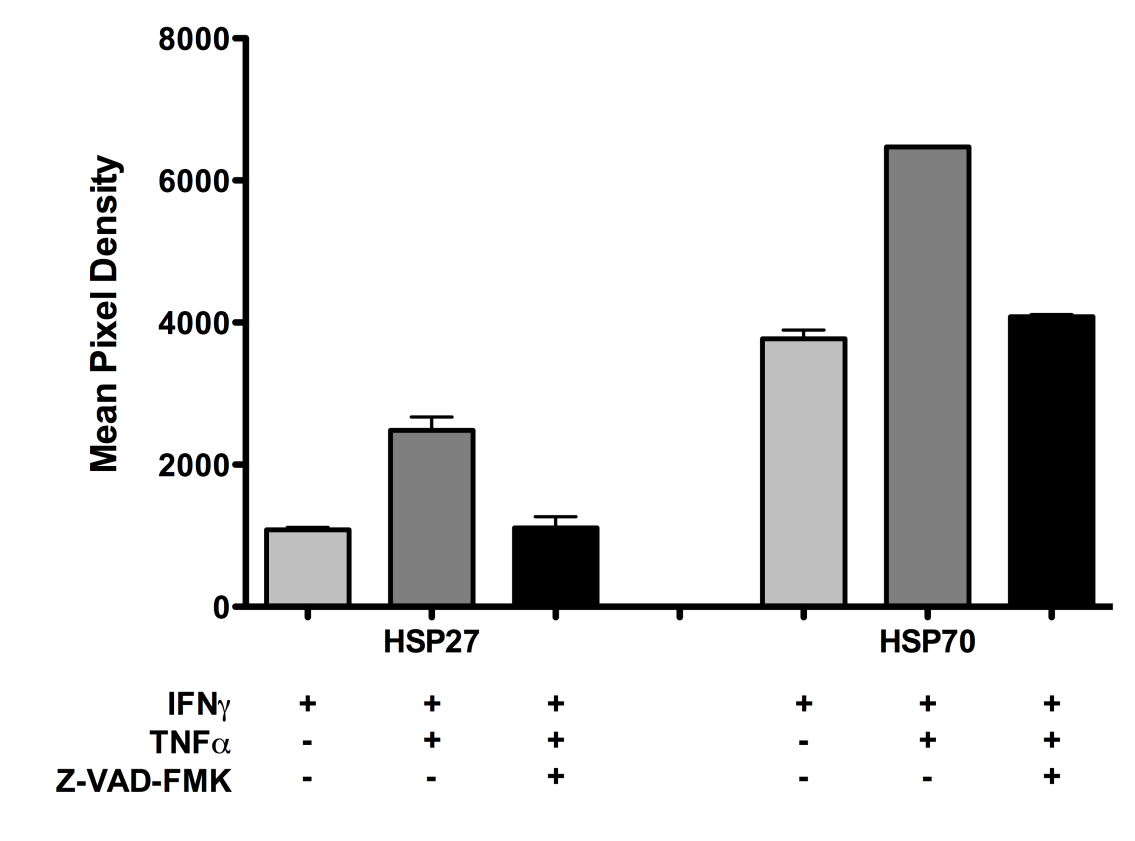

Supplement: Figure S1 — Cytokine treatment of βLox5 cells induces the expression of Heat Shock Proteins. βLox5 cells were treated with the combination of rhTNFα (2000 U/mL) and rhIFNγ (1000 U/mL) for 24 h with and without pan-caspase inhibition with Z-VAD-FMK (50 µM). The Proteome Profiler Human Apoptosis Array Kit was used for protein detection. (TIF) [file pone.0020617.s001.tif]
